# Exercise Capacity in Children With Isolated Congenital Complete Atrioventricular Block: Does Pacing Make a Difference?

**DOI:** 10.1007/s00246-012-0176-0

**Published:** 2012-02-14

**Authors:** A. Christian Blank, Sara Hakim, Jan L. Strengers, Ronald B. Tanke, Toon A. van Veen, Marc A. Vos, Tim Takken

**Affiliations:** 1 Department of Pediatric Cardiology, Wilhelmina Children’s Hospital, University Medical Center, P.O. Box 85090, 3508 AB Utrecht, The Netherlands; 2 Division of Heart and Lungs, Department of Medical Physiology, University Medical Center, Yalelaan 50, 3584 CM Utrecht, The Netherlands; 3 Department of Pediatric Cardiology, University Medical Center St. Radboud, P.O. Box 9101, 6500 HB Nijmegen, The Netherlands; 4 Child Development and Exercise Center, Wilhelmina Children’s Hospital, University Medical Center, P.O. Box 85090, 3508 AB Utrecht, The Netherlands

**Keywords:** Exercise test, Exercise tolerance, Congenital defects, Atrioventricular block

## Abstract

The management of patients with isolated congenital complete atrioventricular block (CCAVB) has changed during the last decades. The current policy is to pace the majority of patients based on a variety of criteria, among which is limited exercise capacity. Data regarding exercise capacity in this population stems from previous publications reporting small case series of unpaced patients. Therefore, we have investigated the exercise capacity of a group of contemporary children with CCAVB. Sixteen children (mean age 11.5 ± 4; seven boys, nine girls) with CCAVB were tested. In 13 patients, a median number of three pacemakers were implanted, whereas in three patients no pacemaker was given. All patients had an echocardiogram and completed a cardiopulmonary cycle exercise test. Exercise parameters were determined and compared with reference values obtained from healthy Dutch peers. The peak oxygen uptake/body mass was reduced to 34.4 ± 9.5 ml kg^−1^ min^−1^ (79 ± 24% of predicted) and the ventilatory threshold was reduced to 52 ± 17% of peak oxygen uptake (78 ± 21% of predicted), whereas the peak work load/body mass was 2.8 ± 0.6 W/kg (91 ± 24% of predicted), which was similar to controls. Importantly, 25% of the paced patients showed upper rate restriction by the pacemaker. In conclusion, children with CCAVB show a reduced peak oxygen uptake and ventilatory threshold, whereas they show normal peak work rates. This indicates that they generate more energy during exercise from anaerobic energy sources. Paced children with CCAVB do not perform better than unpaced children.

## Introduction

Isolated congenital complete atrioventricular block (CCAVB) is a rare cardiac disorder with an estimated incidence of 1/15,000–20,000 live births [27]. In most cases it occurs after damage of a normally structured fetal heart by maternal autoantibodies against ribonucleoproteins (anti-Ro/SSA, anti-La/SSB) [24]. Along with the congenital antibody-associated AV block, a variety of congenital forms of AV block occur secondary to other congenital cardiac defects [25].

Management of patients with CCAVB has changed during the last decennia. In the past, a minority of patients received a pacemaker whereas the current policy is to pace the majority of patients based on a variety of criteria, among which is limited exercise capacity [10].

Exercise capacity provides relevant information about the health status and the ability to perform age-appropriate activities. Furthermore, it is a known predictor of mortality in both healthy and diseased individuals, including patients with congenital heart disease [8, 9, 14, 15].

Data regarding exercise capacity in CCAVB stems from decades-old publications reporting small case series of unpaced patients [31, 32]. Although the current policy is to pace patients, it is unknown whether exercise capacity benefits from this approach.

Therefore, the aim of this cross-sectional study was to investigate the cardiopulmonary exercise capacity of a group of contemporary children with CCAVB with and without pacemaker.

## Methods

### Study Population

The databases of the two participating departments of pediatric cardiology (Utrecht and Nijmegen) were screened to identify all patients >5 years old with isolated CCAVB, which was classified as congenital if (1) CAVB was diagnosed in utero, at birth, or within the neonatal period (0 to 27 days after birth) as proposed by Brucato et al. [6] or (2) CAVB was diagnosed in early childhood without signs and findings of a specific etiology (as described by Yater et al. [46]). The diagnosis “isolated CCAVB” required the absence of major structural heart defects. Eighteen patients were identified, and 16 of them consented to participate in the study. The medical records of those participating patients were reviewed. Data collected included patient age at diagnosis, maternal antibody status, patient age at first pacemaker implantation, all pacemaker-related interventions, and patient status at follow-up.

### Fitness Questionnaire

To obtain information on self-perceived fitness and health, physical activity in daily life, including sports, participation at school, and leisure, a questionnaire [4, 11] from the Department of Integrative Physiology, St. Radboud University Nijmegen Medical Centre, Nijmegen, The Netherlands, was used. The results were compared with those from healthy control subjects [11].

### Anthropometry

Before the cardiopulmonary exercise test (CPET), anthropometric measurements were completed in all patients, including body mass (BM [kg]) and body height (m) using an electronic scale (Seca, Hamburg, Germany) and a stadiometer (Ulmer Stadiometer, Ulm, Germany), respectively. Body mass index (BMI [kg m^−2^]) was calculated as BM in kg divided by the square of the body height in meters. SD scores were calculated for BM for age, body height for age and BMI for age using reference values from the 1997 Dutch Growth Study [12, 13]. To estimate body surface area (BSA [m^2^]), the equation of Haycock et al. was used [19], which has been validated in infants, children, and adults.

### Echocardiography

Before CPET all patients underwent transthoracic echocardiography, which was performed by the same pediatric cardiologist (A. C. B.) with the patient at rest in supine position using a Vivid 7 machine (GE Vingmed Ultrasound AS, Horten, Norway). Images were obtained using a 3.5- or 5.0-MHz transducer in the suprasternal, parasternal, and apical views. Cine-loops, including three cardiac cycles, were stored digitally and analyzed off-line using EchoPac version 7.0.0 software (GE Healthcare, Horten, Norway). After a brief assessment of the cardiac anatomy, the following measurements were performed: M-mode; pulsed-wave (PW) Doppler of the aortic, pulmonic, mitral, and tricuspid valves and descending aorta; PW tissue Doppler imaging (TDI) of the interventricular septum and left free wall; and color-coded TDI of the left ventricle.

### Assessment of Left-Ventricular Size and Function

Parasternal M-mode images were used to measure left-ventricular (LV) end-diastolic (LVEDD) and end-systolic diameters (LVESD). The LVEDD was compared with the normal values of body weight-matched children [26] and expressed as percentage of normal. LV dilatation was defined as LVEDD ≥120% of normal.

Color-coded TDI of the left ventricle in the apical four-chamber view was used to measure the peak systolic and diastolic tissue velocity of the septal and lateral mitral valve (MV) annulus. The velocities were compared with values obtained in a group of healthy young individuals [17]. Abnormal LV function was defined as measured systolic and/ or diastolic velocities <1 SD of the mean velocities of healthy young individuals [17].

### Assessment of Dyssynchrony

Two types of dyssynchrony were assessed: interventricular dyssynchrony and intraventricular dyssynchrony of the left ventricle. Interventricular dyssynchrony was examined by calculating the interventricular mechanical delay (IVMD) using PW Doppler measurements in the left-ventricular (LVOT) and right-ventricular outflow tracts (RVOT) according to the following formula: (time from the onset of QRS to the onset of PW curve in the LVOT)—(time from the onset of QRS to the onset of PW curve in the RVOT). Interventricular dyssynchrony was defined as being present if IVMD >40 ms. Intraventricular dyssynchrony of the left ventricle was assessed by analyzing color-coded TDI of the left ventricle in the apical four-chamber view according to the recommendations of the American Society of Echocardiography Dyssynchrony Writing Group [18]. Intraventricular dyssynchrony was defined as being present if the septal-to-lateral wall delay of the left ventricle was >65 ms.

### CPET

Subjects performed a CPET using an electronically braked cycle ergometer (Ergoline 9000; Ergoline GmbH, Bitz, Germany) as recently described [5]. In short, patients performed a CPET according to the Godfrey protocol [16]. The end of the CPET was marked by symptom limitation. A 12-lead electrocardiogram (ECG) and pulse oximetry (Nellcor 200E; Nellcor, Breda, The Netherlands) were recorded continuously throughout the entire test. Blood pressure was measured every 2 min (SunTech Tango+; SunTech Medical, Morrisville, NC, USA) [36]. The CPET featured a breath-by-breath gas-exchange analysis using a calibrated expiratory gas analysis system (Oxycon Pro; Cardinal, Houten, The Netherlands). Peak values were defined as the highest mean value of any 30 s time interval during exercise. Predicted values were obtained from established values from age- and sex-matched Dutch controls [38].

### Ventilatory Threshold (VT)

The VT was determined using the criteria of an increase in both the ventilatory equivalent of oxygen (VE/VO_2_) and end-tidal pressure of oxygen (P_ET_O_2_) with no increase in the ventilatory equivalent of carbon dioxide (VE/VCO_2_) [7, 44]. P_ET_O_2_ and P_ET_CO_2_ were taken into account to differentiate lactate buffering from hyperventilation. This method has been validated in pediatric patients [30]. VT was expressed as a percentage of predicted VO_2peak_ [35]. Predicted values were obtained from established values from age- and sex-matched Dutch controls [38].

### VE/VCO_2_-Slope and Oxygen Uptake Efficiency Slope (OUES)

The VE/VCO_2_-slope was calculated by linear least-squares regression of the relation between VE and VCO_2_, respectively, during the entire CPET [38]. The OUES was calculated by a linear least-squares regression of the VO_2_ on the common logarithm of the VE, by using the following equation: VO_2_ = *a* logVE + *b* [1]. In this equation, the constant “*a*” stands for the regression coefficient (called the OUES), and “*b*” represents the intercept. Predicted VE/VCO_2_-slope and OUES values were obtained from established values of age- and sex- matched Dutch controls [38].

### Analysis of CPET ECG

During CPET, an electrocardiogram (ECG) was recorded at a speed of 25 mm/s, a gain of 10 mm/mV, and muscle filter +50 Hz. Each minute, a 12-lead trace of several consecutive heartbeats was printed on paper. The traces were analyzed by the same pediatric cardiologist (A. C. B.), who measured the following parameters: rhythm, QRS morphology, QRS duration, and corrected QT interval (QTc) (in normal QRS duration) or corrected JT interval (JTc) (if QRS duration >+2SD). In addition, the presence of arrhythmic events, including pacemaker upper rate behavior (2:1 block or pseudo-Wenckebach), was assessed during exercise.

### Statistical Analysis

Continuous variables are presented as mean ± SD with minimum and maximum. Nominal data are summarized as frequencies and percentages. Differences between patients and reference values were tested using one-sample Student *t* test. Differences between the paced and unpaced group were analyzed using independent-samples Student *t* test, and *p* < 0.05 was considered statistically significant. Statistical analysis was performed with IBM SPSS Statistics 18 for Mac (IBM SPSS, Chicago, IL, USA).

## Results

### Patient Characteristics

The characteristics of the 16 studied patients are listed in Table 1. Patients were diagnosed with CCAVB at a mean age of 0.2 ± 0.6 years. Maternal antibodies were detected at diagnosis in 12 patients (75%). Thirteen patients (81%) had a pacemaker implanted with a median number of three implanted pacemakers. Their first pacemaker was implanted at a mean age of 2.2 ± 4.1 years. Five patients (31%) had minor associated congenital heart defects, such as patent arterial duct (PDA = 2 patients [13%]), PDA and atrial septal defect (PDA + ASD = 1 patient [6%]), ventricular septal defect (VSD = 1 patient [6%]), or ASD and VSD (one patient [6%]). Two patients (13%) were on cardiac medication: one patient used an angiotensin-converting enzyme (ACE) inhibitor, and one patient used a combination of an ACE-inhibitor and a beta-blocker.

**Table 1:** Patient characteristics

Variable	CCAVB group (*n* = 16)	Paced group (*n* = 13)	Unpaced group (*n* = 3)
Male (%)	7 (44)		
Female (%)	9 (56)		
Age at diagnosis (year)	0.2 ± 0.6^a^		
PM (%)			
No PM	3 (19)		
PM	13 (81)		
1st		3 (23)	
2nd		4 (31)	
3rd		1 (8)	
4th		3 (23)	
5th		2 (15)	
Type of pacemaker (%)			
VVIR		3 (23)	
DDD		5 (38)	
DDDR		3 (23)	
CRT		2 (15)	
Programmed upper rate (bpm)		182 ± 11^a^	
Age at first PM implantation (year)		2.2 ± 4.1^a^	
Maternal antibodies (%)			
Negative	3 (20)		
Positive	12 (75)		
Minor associated CHD (%)			
No	11 (69)		
Yes	5 (31)		
ASD/SD	1 (6)		
VSD	1 (6)		
PDA	2 (13)		
PDA/ASD	1 (6)		
Medication (%)			
No	14 (88)		
Yes	2 (13)		
ACE-I	1 (6)		
ACE-I + BB	1 (6)		
Age at CPET (y)	11.5 ± 4.1^a^	12.3 ± 3.9^a^	7.8 ± 2.8^a^
BM (kg)	40.2 ± 16.3^a^	43.9 ± 15.6^a^	23.9 ± 6.2^a^
BM for age (SD)	−0.3 ± 0.9^a^	−0.1 ± 0.9^a^	−1.0 ± 0.4^a^
Body height (m)	1.47 ± 0.21^a^	1.52 ± 0.19^a^	1.24 ± 0.12^a^
Body height for age (SD)	−0.4 ± 0.9^a^	−0.2 ± 0.9^a^	−1.0 ± 0.7^a^
BMI (kg/m^2^)	17.8 ± 3.0^a^	18.4 ± 3.0^a^	15.3 ± 0.9^a^
BMI for age (SD)	−0.1 ± 1.0^a^	0.0 ± 1.1^a^	−0.5 ± 0.1^a^
BSA (m^2^)	1.26 ± 0.34^a^	1.35 ± 0.32^a^	0.90 ± 0.16^a^

*ACE-I* angiotensin-converting enzyme inhibitor, *CHD* congenital heart disease, *BB* beta blocker, *BM* body mass, *BMI* body mass index, *BSA* body surface area, *PM* pacemaker

^a^Data expressed as mean ± SD

### Self*-*Rated Fitness, Health, and Physical Activity

The results of the questionnaire, as listed in Table 2, identified no significant difference in the self-rated fitness and health of paced and unpaced patients. The majority of CCAVB patients were always able to perform physical activities without or with minor difficulties. Most of the CCAVB patients judged their physical condition to be equal to the average of their peers.

**Table 2:** Physical fitness questionnaire

Variable	CCAVB group (*n* = 16)	Paced group (*n* = 13)	Unpaced group (*n* = 3)
Self-rated health (scale 1–10)	8.3 ± 1.1^a^	8.2 ± 1.1^a^	9.0 ± 0.0^a^
Self-rated fitness (scale 1–10)	7.5 ± 1.6^a^	7.3 ± 1.6^a^	8.3 ± 1.5^a^
Participation in sports possible? (%)			
Yes, always, without problems	5 (31)	3 (23)	2 (67)
Yes, always, with some problems	7 (44)	6 (46)	1 (33)
Yes, but not always motivated	3 (19)	3 (23)	
No	1 (6)	1 (8)	
All activities possible in physical education class? (%)			
Yes	9 (56)	6 (46)	3 (100)
No	6 (38)	6 (46)	
Self-rated condition compared with class mates (%)			
Equal to mean of class	14 (88)	11 (85)	3 (100)
Less than mean of class	1 (6)	1 (8)	
Would your call yourself a sportsmen? (%)			
Yes, I’m a real sportsmen	1 (6)	1 (8)	1 (33)
Yes, I do a lot of sports	3 (19)	2 (15)	2 (67)
A bit	9 (56)	7 (540	
No, not really	1 (6)	1 (8)	
No, absolutely not	2 (13)	2 (15)	
Way to school (%)			
Walking	4 (25)	3 (23)	1 (33)
Bicycle	10 (63)	8 (62)	2 (67)
Car	2 (13)	2 (15)	

^a^Data expressed as mean ± SD

### Analysis of LV Size, Function, and Dyssynchrony

The echocardiographic results are listed in Table 3. LVEDD of the studied patients was 110 ± 10% of normal. Three patients (19% [two paced and one unpaced]) had LV dilatation. Two patients (13% [both paced]) showed systolic and diastolic LV dysfunction. MV E velocity (0.99 ± 0.15 vs. 1.39 ± 0.08 m/s, *p* = 0.001), aortic valve (AoV) velocity (1.17 ± 0.13 vs. 1.60 ± 0.03 m/s, *p* < 0.0005), and color-coded TDI lateral MV E’ velocity (−7.8 ± 1.2 vs. −10.7 ± 0.7 cm/s, *p* = 0.002) were significantly higher in unpaced patients. Eight patients (50% [all paced]) showed interventricular dyssynchrony and one patient (6% [paced]) showed intraventricular dyssynchrony.

**Table 3:** Echocardiographic and ECG data

Variable	CCAVB group (*n* = 16)	Comparison paced vs. unpaced	
		Paced group (*n* = 13)	Unpaced group (*n* = 13)
LVEDD % norm	110 ± 10^a^	108 ± 10^a^	117 ± 11^a^
LV dilatation (LVEDD ≥ 120 %norm) (%)	3 (19)	2	1
MV E velocity (m/s)	1.07 ± 0.21^a^	0.99 ± 0.15^a^	1.39 ± 0.08*^,a^
AoV velocity (m/s)	1.25 ± 0.21^a^	1.17 ± 0.13^a^	1.60 ± 0.03*^,a^
Color-coded TDI septal MV S (cm/s)	4.6 ± 0.8^a^	4.5 ± 0.9^a^	5.2 ± 0.1^a^
Color-coded TDI septal MV E′ (cm/s)	−8.4 ± 1.6^a^	−7.8 ± 1.2^a^	−10.7 ± 0.7*^,a^
Color-coded TDI lateral MV S (cm/s)	5.9 ± 1.8^a^	5.6 ± 1.9^a^	7.0 ± 0.3^a^
Color-coded TDI lateral MV E′ (cm/s)	−12.2 ± 3.2^a^	−11.9 ± 3.4^a^	−13.7 ± 0.8^a^
LV systolic dysfunction (%)	2 (13)	2	0
LV diastolic dysfunction (%)	2 (13)	2	0
IVMD (ms)	31 ± 38^a^	42 ± 34^a^	16 ± 2^a^
Interventricular dyssynchrony (%)	8 (50)	8	0
Color TVI septal-lateral delay (ms)	18 ± 23^a^	18 ± 26^a^	20 ± 10^a^
Intraventricular dyssynchrony (%)	1 (6)	1	0
Rest QRS duration (ms)	136 ± 23^a^	145 ± 13^a^	97 ± 6*^,a^
QRS morphology (%)			
LBBB-like	11 (69)	11	3
IRBBB	3 (19)		
Indifferent	2 (13)	2	
Rest QTc (unpaced subjects) (ms)	420 ± 18^a^	NA	420 ± 18^a^
Rest JTc (paced subjects) (ms)	311 ± 24^a^	311 ± 24^a^	NA
PVBs (%)	0 (0)	0	0
Upper rate behavior (paced subjects) (%)	4 (25)	4	NA

*IRBBB* incomplete right bundle branch block, *LBBB* left bundle branch block

* *p* < 0.05

^a^Data presented as mean ± SD

### CPET ECG

Table 3 lists the ECG results. QRS duration during the rest phase of CPET (145 ± 13 vs. 97 ± 6 ms, *p* < 0.0005) was significantly shorter in unpaced patients. Eleven of the (RV) paced patients showed an LBBB-like QRS morphology. Two of the (cardiac resynchronization therapy [CRT]) paced patients had an indifferent QRS morphology. The JTc in paced patients was 311 ± 24 ms (normal value < 350 ms). The QTc in unpaced patients was 420 ± 18 ms (normal value < 440 ms). Despite of an average programmed pacemaker upper rate of 182 ± 11 bpm, four patients (25% [three DDDR (dual [atrial/ventricular] paced, dual [atrial/ventricular] sensed, dual [inhibited/triggered], rate responsive) paced and one CRT paced]) showed upper rate behavior of their pacemaker (one pseudo-Wenckebach and three 2:1 block). No patient displayed premature ventricular beats.

### CPET

Table 4 lists the CPET data. Peak HR (135 ± 37 bpm), VO_2peak_ (1.31 ± 0.50 l min^−1^ [79 ± 24% of predicted]), VO_2peak_ kg^−1^ (34.4 ± 9.5 ml kg^−1^ min^−1^ [79 ± 24% of predicted]), peak minute ventilation (50.2 ± 22.9 l min^−1^ [76 ± 26% of predicted]), and VT (52 ± 17% of VO_2peak_ [78 ± 21% of predicted]) were all significantly lower than those of healthy peers.

**Table 4:** CPET data

Variable	Comparison CCAVB vs. healthy peers [38] (*p* < 0.05)*	Comparison paced vs. unpaced	
	CCAVB group (*n* = 16)	Paced group (*n* = 13)	Unpaced group (*n* = 3)
Rest HR (bpm)	73 ± 13^a^	77 ± 10^a^	53 ± 2*^,a^
Peak HR (bpm) (% predicted)	135 ± 37^a^ (70 ± 19)*^,a^	139 ± 35^a^ (72 ± 18)^a^	117 ± 48^a^ (60 ± 25)^a^
VVIR		114 ± 25^a^	
DDD		171 ± 8^a^	
DDDR		98 ± 6^a^	
CRT		161 ± 8^a^	
Rest VO_2_ (l min^−1^)	0.28 ± 0.07^a^	0.29 ± 0.08^a^	0.23 ± 0.04^a^
Peak RER	1.13 ± 0.11^a^	1.15 ± 0.12^a^	1.06 ± 0.01^a^
Peak workload/BM (W kg^−1^) (% predicted)	2.8 ± 0.6^a^ (91 ± 24)^a^	2.8 ± 0.6^a^ (87 ± 23)^a^	3.3 ± 0.2^a^ (112 ± 11)^a^
Peak VO_2_ (l min^−1^) (% predicted)	1.31 ± 0.50^a^ (79 ± 24)*^,a^	0.95 ± 0.12^a^ (68 ± 23)^a^	1.39 ± 0.51^a^ (112 ± 39)^a^
Peak VO_2_/BM (in ml kg^−1^ min^−1^) (% predicted)	34.4 ± 9.5^a^ (79 ± 24)*^,a^	33.0 ± 9.5^a^ (75 ± 24)^a^	40.5 ± 8.3^a^ (93 ± 22)^a^
Peak VE (l) (% predicted)	50.2 ± 22.9^a^ (76 ± 26)*^,a^	52.4 ± 24.8^a^ (72 ± 27)^a^	40.7 ± 8.1^a^ (92 ± 19)^a^
VT% peak VO_2_ (% predicted)	52 ± 17^a^ (78 ± 21)*^,a^	51 ± 18^a^ (79 ± 22)^a^	53 ± 14^a^ (73 ± 15)^a^
VO_2_/BM at VAT (ml kg^−1^ min^−1)^	22.9 ± 6.2^a^	22.0 ± 6.0^a^	26.9 ± 6.6^a^
O_2_ pulse (ml beat^−1^) (% predicted)	10.4 ± 5.1^a^ (111 ± 56)^a^	10.8 ± 5.6^a^ (98 ± 31)^a^	8.6 ± 1.7^a^ (170 ± 106)^a^
Peak systolic BP (mmHg) (% predicted)	131 ± 24^a^ (99 ± 12)^a^	130 ± 26^a^ (84 ± 11)^a^	137 ± 15^a^ (98 ± 6)^a^
VE/VCO_2_ slope (% predicted)	33.3 ± 6.6^a^ (108 ± 17)^a^	31.8 ± 6.3^a^ (105 ± 18)^a^	40.0 ± 1.8*^,a^ (121 ± 2)^a^
OUES (% predicted)	1424 ± 510^a^ (92 ± 39)^a^	1513 ± 528^a^ (81 ± 23)^a^	1034 ± 57*^,a^ (141 ± 60)*^,a^
FEV1% predicted	92 ± 11^a^	92 ± 12^a^	89 ± 5^a^
FVC% predicted	87 ± 15^a^	87 ± 16^a^	87 ± 13^a^
Peak oxygen saturation (%)	96 ± 2^a^	96 ± 3^a^	97 ± 1^a^

*RER* respiratory exchange ratio, *O*_*2*_ *pulse* peak VO_2_/peak HR, *BP* blood pressure

* (*p* < 0.05)

^a^Data presented as mean ± SD

Peak work load corrected for body mass (2.8 ± 0.6 W kg^−1^ [91 ± 24% of predicted]), peak oxygen pulse (10.4 ± 5.1 ml beat^−1^ [111 ± 56% of predicted]), peak systolic blood pressure (131 ± 24 mmHg [99 ± 12% of predicted]), VE/VCO_2_-slope (33.3 ± 6.6 [108 ± 17% of predicted]), OUES (1424 ± 510 [92 ± 39% of predicted]), percentage of predicted FEV_1_ (92 ± 11%), percentage of predicted forced expiratory vital capacity (FVC) (87 ± 15%), and peak SpO_2_% (96 ± 2%) differed nonsignificantly from healthy peers.

**Fig. 1:**
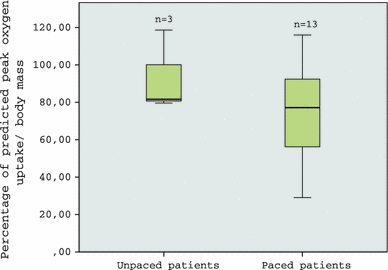
Percentage of predicted peak oxygen uptake/body mass (VO_2peak_ kg^−1^) of unpaced and paced CCAVB patients. Box-and-whisker diagram: *horizontal line* in the *box* depicts median value; the *box includes* 50% of the values; the *upper whisker* represents the top 25% of the values; and the *lower whisker* represents the bottom 25% of the values

**Fig. 2:**
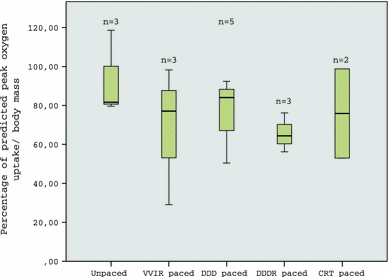
Percentage of predicted peak oxygen uptake/body mass (VO_2peak_ kg^−1^) of unpaced and paced CCAVB patients. The paced patients are subdivided according to their pacemaker type. *Box*-and-*whisker* diagram: *horizontal line* in the *box* depicts median value; the *box* includes 50% of the values; the *upper whisker* represents the top 25% of the values; and the *lower whisker* represents the bottom 25% of the values

Paced CCAVB patients showed a significantly higher HR at rest (77 ± 10 vs. 53 ± 2 bpm), lower VE/VCO_2_-slope (31.8 ± 6.3 vs. 40.0 ± 1.8) and higher OUES (1513 ± 528 vs. 1034 ± 57) compared with unpaced patients. There were no significant differences in all other CPET variables between paced and unpaced patients. There were no significant differences in VO_2peak_ kg^−1^ between paced and unpaced patients (Fig. 1) and between the various pacemaker types (Fig. 2). Peak HR of the paced patients differed between the various pacemaker types. DDD (dual [atrial/ventricular] paced, dual [atrial ventricular] sensed, dual [inhibited/triggered])-paced patients had the highest peak HR (171 ± 8 bpm), followed by CRT (161 ± 8 bpm), VVIR (ventricular paced, ventricular sensed, inhibited, rate responsive) (114 ± 25 bpm), and DDDR-paced (98 ± 6 bpm) patients.

## Discussion

The aim of this cross-sectional study was to investigate the cardiopulmonary exercise capacity of contemporary children with CCAVB with and without pacemaker. Our study showed that VO_2peak_ kg^−1^ is significantly decreased in children with CCAVB. The VO_2peak_ kg^−1^ values (34.4 ± 9.5 ml kg^−1^ min^−1^) are comparable with those observed approximately 30 years ago in unpaced children with CCAVB [29, 40, 43]. These studies reported an average VO_2peak_ kg^−1^ of 36 ± 2, 37, and 31 ml kg^−1^ min^−1^, respectively.

Furthermore, the VO_2_ at VT was significantly decreased compared with healthy peers. The observed VO_2_ at VT (22.9 ± 6.2 ml kg^−1^ min^−1^) was comparable with the VT observed in unpaced children with CCAVB as reported >20 years ago by Reybrouck et al. (22.8 ± 5.5 ml kg^−1^ min^−1^) [32].

It is of interest to note that the peak workload corrected for body mass was not significantly decreased in children with CCAVB. This implies that children with CCAVB are generating more energy from anaerobic sources during exercise, compared with healthy peers, as a compensatory mechanism for their decreased cardiac output. Furthermore, this explains why children with CCAVB do not frequently report exercise intolerance [2]. Based on these figures, the question arises whether pacemaker therapy globally improves exercise capacity in children with CCAVB. Unexpectedly, our current results suggest that the VO_2peak_ and VT values of our paced patients do not differ from those obtained in unpaced patients from 20 to 30 years ago. Moreover, VO_2peak_ values between the paced and unpaced patients of our study population were not significantly different.

Our hypothesis is that by employing the current indications for pacing in CCAVB, as reviewed by Villain [41], only the “best” patients in terms of exercise capacity stay unpaced. Apparently, the exercise capacity of these “best” unpaced patients can compete with the paced patients. Thus, perhaps there are factors that prevent normalization of exercise capacity after insertion of a pacemaker.

VO_2peak_ is regarded as the single best parameter to describe exercise capacity [34]. According to the Fick equation, VO_2peak_ is the product of three parameters: peak heart rate (HR_peak_), peak stroke volume (SV_peak_), and peak arterial-venous oxygen difference (CaO_2_ − CvO_2_); (VO_2peak_ = HR_peak_ × SV_peak_ × (CaO_2_ − CvO_2_)). Dynamic changes in one of these parameters related to exercise, therefore, might influence the oxygen transport during exercise.

### Peak Heart Rate

Unpaced children with CCAVB mostly have an AV junctional escape rhythm with a lower frequency than healthy individuals at rest (average 46 [32] to 59 [37] bpm [in this study 53 bpm]). In these patients, this frequency approximately doubles (range 1.6 [37] to 2.3 [40]) during (peak) exercise to an average frequency of 94 [37] to 117 [in this study]). A pacemaker will restore the frequencies at rest as well as during exercise. The extent of frequency restoration depends on the pacemaker mode (e.g., single chamber [VVI] vs. dual chamber (DDD), use of rate response) and the pacemaker programming. Our study showed that the average peak HR of paced CCAVB patients is still lower than in healthy individuals. There are two reasons for that finding. First, approximately half of the paced patients had a rate-responsive pacemaker (i.e., VVIR or DDDR). They showed a lower average peak HR compared with DDD- or CRT-paced patients. This means that the sensor sensitivity was not appropriate for the type of exercise (cycling). All rate-responsive pacemakers in our study used an accelerometer as activity sensor. Theoretically, an accelerometer should be sensitive to bicycling because it detects horizontal movement [42]. However, it is designed for use in adult patients with the pacemaker implanted in the pectoral region [3]. The majority of patients with a rate-responsive pacemaker in our study had their device implanted in the abdominal region, which blunted the response of their accelerometer during bicycling. Our results underscore the necessity to use the treadmill instead of the bicycle for the exercise testing of pediatric patients with a rate-responsive pacemaker.

Second, despite an average programmed pacemaker upper rate (maximum tracking rate [MTR]) 182 ± 11 bpm minute, 25% of the paced patients showed upper rate behavior of the pacemaker during the CPET, which limited their exercise capacity significantly. These results show that pediatric CCAVB patients would benefit from MTRs > 180 bpm. Unfortunately, not all pacemaker models and manufacturers support MTRs in that range. Finally, another factor that might influence peak HR is the occurrence of premature ventricular beats (PVBs). Earlier studies showed a high incidence (27% [22] to 70% [45]) of PVBs in unpaced CCAVB patients. However, no patient in our study displayed PVBs.

### Peak Stroke Volume

The stroke volume is influenced by cardiac preload, myocardial contractility, and cardiac afterload. There is a difference in these parameters between unpaced and paced CCAVB patients. In unpaced patients, according to Scarpelli and Rudolph [33], the long diastolic filling in bradycardia causes an increased end-diastolic volume with stretching of the myocardial fibres, augmenting myocardial contractility. Indeed, Kertesz et al. [23] demonstrated that moderate LV dilatation is common in these patients and is associated with a normal LV geometry, normal wall stress, and enhanced systolic function during the first two decades of life. The data of this study support the findings of Scarpelli and Rudolph and Kertesz et al. The LV end diastolic diameter was greater (although not significantly) in unpaced patients, and one patient had LV dilatation. The MV Doppler inflow velocity was significantly greater in unpaced patients, suggesting an unfavorable relaxation of the stretched left ventricle. All unpaced CCAVB patients had normal LV function.

Paced CCAVB patients had a lower LVEDD and MV Doppler inflow velocity, suggesting less stretching and better relaxation of the left ventricle. However, two of 13 paced patients (15%) showed LV dysfunction (with LV dilatation in one patient). This might be a result of chronic pacing. Earlier studies have shown that chronic (right) ventricular pacing causes an abnormal electrical activation that may lead to mechanical dyssynchrony (seen in 62% of the paced patients in this study), LV remodeling, LV dilatation, LV dysfunction, and low exercise capacity [28, 39].

The majority of our patients (87%) had normal LV function at rest. Yet, stroke volume can increase as well as decrease during exercise in the presence of normal LV function at rest [20, 21, 37]. Therefore, future studies should include exercise echocardiography with (noninvasive) measurement of stroke volume.

### Arterial–Venous Oxygen Difference

Although our study does not include (invasive) measurement of the oxygen content of the arterial and venous blood, earlier studies showed a normal (13.8 [43] to 14.5 [29] ml/100 ml) or increased [21] average arterial–venous oxygen difference during (peak) exercise in patients with CCAVB. It is therefore unclear whether children with CCAVB have an increased arterial–venous oxygen difference during exercise as a compensatory mechanism for their decreased cardiac output.

### Study Limitations

A limitation of this study was that our data were obtained cross-sectionally from a rather small group of patients in two University Children’s Hospitals. In contrast, all patients were tested by the same experienced staff using the same equipment to avoid interobserver or technical variability. Future additional studies should be performed in a larger patient population and should preferably include more unpaced CCAVB patients (although lack of availability is a limitation in itself). In addition, longitudinal exercise data are desirable for investigating the effects of pacemaker therapy on exercise capacity in this population.

In conclusion, children with CCAVB show a decreased peak oxygen uptake and VT, whereas they show normal peak work rates. This indicates that they generate more energy from anaerobic energy sources during exercise. Paced children with CCAVB do not perform better than unpaced children. Possible explanations for that finding might be: (1) a selection bias imposed by the current pacemaker criteria (only the clinically “best” CCAVB patients stay unpaced), (2) chronic RV pacing–induced LV dysfunction in some CCAVB patients, and (3) suboptimal pacemaker programming.

Future exercise studies, including a greater number of patients and longitudinal follow-up, are warranted to investigate the influence of myocardial properties (LV dysfunction), pacing mode, and optimal pacemaker programming on exercise capacity.
